# Transactions of the Pathological Society of Philadelphia

**Published:** 1840-03-07

**Authors:** 


### TRANSACTIONS OF THE PATHOLOGICAL SOCIETY OF PHILADELPHIA.

*February 28th, 1840.*

The President, Dr. GERHARD, in the Chair.

### *Case of Fracture of the Spleen.*

BY FRANCIS WEST, M. D.

J. C., a stout, healthy man, 21 years of age, whilst running with a fire engine along Fourth near Market street, was caught between it and a pump, which stood at the side of the street. The blow was received upon his left side, and completely mashed a breast pin which was in his waistcoat pocket. He immediately fell and was carried into a neighbouring tavern. When I saw him about ten minutes after the accident, he was perfectly insensible, pulseless, with a pale, cool skin, his mouth covered with coloured frothy saliva, and his respiration short and singultous. The only external mark of injury was over the left hypochondrium, where the skin was slightly scratched. Pressure beneath the false ribs of this same side caused great uneasiness, evidenced by writhing of the body and alteration of the features. On closer examination something (supposed to be the spleen) could be distinctly felt projecting beyond the ribs, pressure upon which increased the patient's suffering. The abdomen was soft and not much swelled. In a few minutes after I saw him, he threw up some frothy, light coloured blood, and immediately expired.

From his symptoms, and the account given by his friends of the accident, I was led to believe that his spleen was fractured or had suffered some other injury, and so I stated to the coroner's jury.

The coroner thought that some depression existed about the sixth or seventh dorsal vertebra, caused perhaps by luxation of one of them; of this, however, I was not satisfied. The patient was not observed to move his legs after the accident, but whether this was the effect of paralysis, or merely from prostration, it was impossible to decide with certainty. Leave was obtained to examine the body. The spine was laid bare, and as far as we could judge from a superficial examination, made by placing the fingers upon opposite points of the column, no *positive* assurance was given, either of fracture or dislocation of the vertebræ, though it was thought that, *perhaps*, a somewhat unnatural depression did exist at the part indicated. We had not the instruments necessary to pursue the examination to perfect satisfaction.

On opening the abdomen, however, signs of injury were very manifest. In the left hypochondriac region, an immense effusion of blood was found, and on removing the spleen, this organ presented a solution of continuity through its right edge, to the depth of one and a half inches, with several lacerations of its substance upon its under surface.

The other organs of the abdomen and chest were uninjured. The omentum everywhere presented a highly injected, arborescent appearance, of a bright red colour, resembling intense inflammation. The lesion of the spleen, together with the shock given to the system by the accident, were deemed to be sufficient cause of the man's death.

The fracture of so small an organ as the spleen, with so little injury to the neighbouring parts, affords an interesting example of the varied action of external violence upon the internal organs of the body, which, as we know, it is always difficult to estimate with any certainty.

*Philadelphia, March* 2, 1840.

